# Study Selection Bias and Racial or Ethnic Disparities in Estimated Age at Onset of Cardiometabolic Disease Among Midlife Women in the US

**DOI:** 10.1001/jamanetworkopen.2022.40665

**Published:** 2022-11-07

**Authors:** Alexis Reeves, Michael R. Elliott, Tené T. Lewis, Carrie A. Karvonen-Gutierrez, William H. Herman, Siobán D. Harlow

**Affiliations:** 1 Department of Epidemiology, School of Public Health, University of Michigan, Ann Arbor; 2 Department of Biostatistics, School of Public Health, University of Michigan, Ann Arbor; 3 Survey Research Center, Institute for Social Research, University of Michigan, Ann Arbor; 4 Department of Epidemiology, Rollins School of Public Health, Emory University, Atlanta, Georgia; 5 Department of Internal Medicine, Michigan Medicine, University of Michigan, Ann Arbor; 6 Department of Obstetrics and Gynecology, Michigan Medicine, University of Michigan, Ann Arbor

## Abstract

**Importance:**

Racial disparities in cardiometabolic health are consistently observed in cohort studies. However, most studies neither evaluate differences in age at onset nor account for systematic exclusion stemming from “weathering” (accelerated health declines for minoritized groups due to structural social and economic marginalization).

**Objective:**

To evaluate racial or ethnic disparities in age at onset of 4 cardiometabolic outcomes (hypertension, isolated systolic hypertension [ISH], insulin resistance [IR], and diabetes), accounting for multiple forms of potential selection bias.

**Design, Setting, and Participants:**

This cohort study used data from the Study of Women’s Health Across the Nation longitudinal cohort (1995-2016) and a cross-sectional screening sample (1995-1997). Data were analyzed from July 2019 to October 2021. Participants were eligible for the cohort if they were aged 42 to 52 years, had not received hormone therapy in the past 3 months, were not pregnant, had an intact uterus and at least 1 ovary, and were premenopausal or early perimenopausal (most recent menses ≤3 months).

**Exposures:**

Self-reported racial or ethnic group (Black, Chinese, Hispanic, Japanese, or White).

**Main Outcomes and Measures:**

The main outcomes were hypertension (systolic blood pressure [BP] ≥140 mm Hg and diastolic BP ≥90 mm Hg or use of antihypertensive medication), ISH (systolic BP ≥140 mm Hg and diastolic BP <90 mm Hg or use of antihypertensive medication), IR (homeostasis model assessment for IR value >5.9 or insulin use), and diabetes (fasting serum glucose level ≥126 mg/dL [to convert to mmol/L, multiply by 0.0555], use of insulin or oral antidiabetic medication, or physician diagnosis). Selection into the cohort was addressed via inverse probability weighting and interval-censored survival models and selection out via multiple imputation. Accelerated failure time models were used to examine racial or ethnic differences in age at disease onset and estimate the median age at onset.

**Results:**

A total of 3302 women were included in the study, with a median age of 46.2 years (range, 42-52 years) at baseline. In the sample, 42.6% had a bachelor’s degree or higher and 36.3% self-rated their health as “very good” at baseline; 23.9% had hypertension, 43.7% had ISH, 13.5% had IR, and 4.6% had diabetes at baseline. Hypertension occurred a median of 5.0 years (95% CI, 5.4-5.5 years) earlier and metabolic outcomes (diabetes and IR) a median of 11.3 years (95% CI, 9.7-12.9 years) earlier for Black and Hispanic women vs White women; ISH occurred a median of 7.7 years (95% CI, 7.3-8.1 years) earlier for Black women vs White women. Adjustment for selection was associated with a mean 20-year decrease in estimated median age at onset, with greater decreases among Black and Hispanic women.

**Conclusions and Relevance:**

In this multiracial cohort of midlife women, failure to account for selection biases, especially at study onset, was associated with falsely high estimates of age at cardiometabolic onset, with greater misestimation among Black and Hispanic women. The results suggest that hypertension and metabolic interventions, particularly for Black and Hispanic women, should be targeted to women aged as young as 30 years for hypertension and 40 years for metabolic interventions. Considering the timing of disease and fully addressing inherent selection biases in research are critical to understanding aging and disease risk, especially for racial and ethnic minoritized populations.

## Key Points

**Question:**

Is systematic exclusion, or selection bias, into and out of cohort studies associated with underestimation of the magnitude of racial or ethnic disparities in age at onset of cardiometabolic disease?

**Findings:**

In this cohort study of 3302 women, onset of hypertension, insulin resistance, and diabetes occurred earlier in Black and Hispanic women compared with White women. Addressing study selection was associated with a decrease in estimated age at onset, with greater differences among Black and Hispanic vs White women.

**Meaning:**

The findings suggest that fully addressing selection bias in cohort studies is necessary to correctly estimate the magnitude of racial and ethnic health disparities.

## Teaser

This cohort study evaluates the association of selection bias into and out of cohort studies with racial or ethnic disparities in the estimation of age at onset of cardiometabolic disease in a cohort of midlife women in the US.

## Introduction

Racial disparities in cardiometabolic health are consistently observed in cohort studies.^1,2,3,4^ Studies suggest that cardiometabolic risk factors and outcomes such as hypertension, diabetes, stroke, and cardiovascular disease have a higher prevalence at earlier ages in Black vs White populations.^4,5,6,7,8,9^ Despite increasing attention to the issue of “weathering,” or earlier onset of disease for marginalized groups caused by the repeated lifetime experience of social and economic adversity and structural marginalization,^5^ little research has focused on understanding the magnitude of racial and ethnic differences in the age at onset of these conditions.

Furthermore, many cohorts have not accounted for the potential exclusion of racial and ethnic minoritized groups from studies because of weathering, which may lead to underestimation of disparities. In cohort studies, the typical goal is to follow disease-free persons over time to observe disease onset.^10,11^ Consequently, studies typically recruit participants in a particular age range determined by the usual age at onset of the outcome, excluding persons who have the outcome at recruitment. However, failure to adjust recruitment ages to account for the earlier ages at onset in racial and ethnic minoritized populations could systematically exclude populations that experience weathering, thereby leading to selection bias, or a distortion in estimation caused by differential selection into or out of a study or analysis.^12^

This study aimed to assess the magnitude of differences among racial or ethnic groups (Black, Chinese, Hispanic, and Japanese compared with White) in age at onset of 4 cardiometabolic outcomes—hypertension, isolated systolic hypertension (ISH), insulin resistance (IR), and diabetes—in midlife women using the Study of Women’s Health Across the Nation (SWAN), a multiethnic longitudinal cohort study. We corrected for 3 sources of selection stemming from selection into (left-truncation and censoring) and out of (right-censoring) the cohort and examined how selection affects the estimation of racial or ethnic differences in the age at onset of cardiometabolic outcomes that vary with age.^4,13,14,15^

## Methods

### Participants

This cohort study used data from women aged 42 to 52 years in the 1995 to 1997 SWAN cross-sectional screening study (only to create left-truncation weights) and the resulting cohort study, with follow-up until 2015 to 2016.^16^ Data were analyzed from July 2019 to October 2021. The screening study recruited participants via community-based sampling, random-digit telephone dialing, and “snowballing” (participant referral)^16^ to identify women aged 40 to 55 years in the racial or ethnic groups for each site, with survey language tailored to recruit particular racial or ethnic groups for each site (Black: Southeast Michigan; Boston, Massachusetts; Chicago, Illinois; and Pittsburgh, Pennsylvania [English]; Chinese: Oakland, California [English or Cantonese]; Hispanic: Newark, New Jersey [English or Spanish]; Japanese: Los Angeles, California [English or Japanese]; and White: all sites [English]).^16^ Women were eligible for the cohort if they were 42 to 52 years of age, had not received hormone therapy (birth control, fertility drugs, estrogens or progestins, hormone patches or creams, hormone injections, or postmenopausal hormones) in the past 3 months, were not pregnant, had an intact uterus and at least 1 ovary (no hysterectomy and/or oophorectomy), and were premenopausal or early perimenopausal (most recent menses ≤3 months). The study was exempted from approval by the institutional review board at each study site because it used secondary data from SWAN; participants provided written informed consent yearly. This study followed the Strengthening the Reporting of Observational Studies in Epidemiology (STROBE) reporting guideline, although we present only the covariate-adjusted estimates for brevity.

### Racial or Ethnic Groups

Participants self-identified their primary racial or ethnic group. Categories in SWAN included Black (Black or African American individuals or those of African descent), Chinese (those of Chinese origin or Chinese American individuals), Hispanic (Mexican American or Cuban American individuals or those of Puerto Rican, Mexican, Dominican, Central American, Cuban, South American, Spanish, or other Hispanic descent), Japanese (those of Japanese origin or Japanese American individuals), and White.

### Primary Outcomes

Blood pressure (BP) measurements and antihypertensive medication use (verified by review) were obtained at yearly visits (16 measurements). Three seated measurements of BP were taken; the second and third measurements were averaged. Stage 1 and 2 hypertension were assessed as systolic BP of 130 mm Hg or higher and diastolic BP of 80 mm Hg or higher (stage 1), systolic BP of 140 mm Hg or higher and diastolic BP of 90 mm Hg or higher (stage 2), and/or by use of antihypertensive medication.^14^ Stage 1 and 2 ISH were assessed as systolic BP of 130 mm Hg or higher and diastolic BP less than 80 mm Hg (stage 1), systolic BP of 140 mm Hg or higher and diastolic BP less than 90 mm Hg (stage 2), and/or by use of antihypertensive medication. Insulin level was measured in fasting blood samples using a solid-phase radioimmunoassay at approximately yearly visits (11 measurements). The homeostasis model assessment for insulin resistance (HOMA-IR) was used to quantify IR, determined as a HOMA-IR value greater than 5.9^17^ or having insulin-treated diabetes (verified by review). Type 2 diabetes was determined yearly (16 measurements) by 1 or more of the following: 10-hour fasting serum glucose level of 126 mg/dL or higher at 11 visits (to convert to mmol/L, multiply by 0.0555), use of insulin or oral antidiabetic medication (verified by review), or physician diagnosis.

### Covariates

Covariates were chosen based on their associations with race and ethnicity and cardiometabolic disease in prior studies.^18,19^ Educational level was stratified into 3 categories (high school diploma or less, some college or an associate’s degree, or a bachelor’s degree or higher). Health was self-reported on a 5-point scale from 1 (“poor”) to 5 (“excellent”), and answers were collapsed into a 4-level variable (excellent, very good, good, and fair or poor). Waist circumference in centimeters was assessed with a measuring tape placed horizontally at the level of the natural waist or the narrowest part of the torso. Participants self-reported smoking status (never, former, or current smoker). Alcohol intake was collapsed into “none or low” (<2 drinks/week), “moderate” (2-7 drinks/week), and “high” (>7 drinks/week). Physical activity scores ranged from 3 to 15; each 1-unit increase reflected higher intensity or frequency of a self-reported work, leisure, or sport activity.^20^

### Multiple Imputation for Right-Censoring

Right-censoring can occur due to dropout, missing data, and/or exclusion of certain subgroups from analysis. Multiple imputation by chained equations^21^ was used to estimate the values of missing data in follow-up (ie, right-censoring) in 2 steps. First, all covariates (fraction of missing information ≤5%) and the occurrence of each outcome at baseline and follow-up (fraction of missing information ≤40%) (eTable 1 in the Supplement) were imputed (*m* = 10). For each imputation, age at the first occurrence of each outcome was imputed, with previously imputed data incorporated as additional predictors.

### Weighting for Left-Truncation

Left-truncation occurs when individuals who have already had the outcome of interest are not included in the study. For each imputed data set, 3 inverse probability weights^22,23^ were estimated to weight the current cohort to represent the cohort that would have been enrolled if (1) there were no eligibility criteria applied (eligibility weight); (2) once eligible, there was no differential participation (participation weight); and (3) everyone was recruited at age 42 years (study design weight). Data from the screening study (only for women aged 42-52 years) were used for the eligibility and participation weight, calculated as the inverse probability of eligibility, and participation given eligibility. Predictors for each included race or ethnicity, study site, educational level, marital status, hormone use, parity, fibroids, self-reported health, heart attack or angina, osteoporosis, cancer (excluding skin cancer), smoking status, and body mass index, with 2-way interactions between race or ethnicity and educational level. Age was excluded as a predictor to avoid double counting the effect of age (included in the study design weight). Predictors were selected a priori and using forward-backward selection (accuracy, 0.65-0.80). The study design weight was proportional to the 10-year probability of each woman aged 42 to 52 years being excluded from the study given age and eligibility status. Weights were multiplied to simultaneously account for all left-truncation selection mechanisms.

### Statistical Analysis

Means, proportions, and SEs were pooled across the imputation sets for all variables across race or ethnicity groups using the Rubin rules.^21^ Accelerated failure time models were used to estimate the racial or ethnic differences in age at onset (first occurrence) of hypertension, ISH, IR, and diabetes. Left-censoring occurs when those who already have the outcome of interest at study baseline, or prevalent cases, are excluded from analysis. Accelerated failure time models incorporate left-censored cases in a survival model by treating prevalent cases as interval censored, where the outcome occurs between a set interval (in this study, between age 20 years and the baseline age). The lognormal distribution was the best fit to the data; thus, the interpretation of the accelerated failure time estimates was the percentage difference in the mean time to each outcome for each racial or ethnic group (reference, White women). The racial or ethnic differences in time to each outcome were estimated in 4 progressive models: unadjusted for selection (model 1), incorporating left-censored cases (interval-censored model; model 2), model 2 accounting for right-censoring (multiply imputed; model 3), and model 3 adjusted for left-truncation (weighted; model 4). The 95% CIs in weighted models were calculated using Taylor series approximations.^24^ Imputed estimates were pooled using the Rubin rules.^21^ All models adjusted for socioeconomic, behavior, and health covariates.^18,19^ The estimated median age at onset and 95% CIs overall and for each racial or ethnic group were calculated, with the covariate profile set to mean levels. Two-sided *P* < .05 was considered statistically significant. Analyses were conducted in Stata, version 16 (StataCorp LLC),^25^ and R, version 4.2.0 (R Project for Statistical Computing).^26^

## Results

This study used data from 15 695 participants in the SWAN screening study (median age, 46.9 years [range, 42-52 years]) to create left-truncation weights. A total of 3302 women (median age at baseline, 46.2 years [range, 42-52 years]) were included in the cohort study; 934 (28.3%) were Black, 250 (7.6%) were Chinese, 286 (8.7%) were Hispanic, 281 (8.5%) were Japanese, and 1551 (47.0%) were White. At baseline, the plurality of individuals in the cohort sample had a bachelor’s degree or higher (42.6%). Overall, most women rated their health as “very good” (36.3%); among Black and Hispanic women, the largest proportions self-rated their health as “good” (35.7% and 46.2%, respectively). Only 4.7% of the sample used insulin throughout follow-up. Nearly half the sample used antihypertensive medication throughout follow-up (49.4%) (Table 1).

**Table 1.** Baseline Characteristics of Participants in the Study of Women’s Health Across the Nation, 1995 to 1997, Using Multiply Imputed Data

Characteristic	Participants^a^						
	Overall (N = 3302)	Black (n = 934)	Chinese (n = 250)	Hispanic (n = 286)	Japanese (n = 281)	White (n = 1551)	*P* value^b^
Racial or ethnic group, %^c^	100	28.3	7.6	8.7	8.5	47.0	NA
Baseline characteristic							
Age, mean (SE), y^c^	46.3 (0.05)	46.2 (0.09)	46.5 (0.16)	46.3 (0.16)	46.7 (0.16)	46.3 (0.07)	.18
Educational level^d^^,^^e^							
≤High school	25.1	26.8	29.0	72.1	18.3	16.1	<.001
Some college or associate’s degree	32.3	41.4	21.8	18.8	34.3	30.6	
≥Bachelor’s degree	42.6	31.9	49.2	9.1	47.4	53.3	
Financial hardship^d^							
Very hard	9.3	12.5	5.2	26.4	3.6	6.0	<.001
Somewhat hard	30.7	33.8	22.9	55.1	26.7	26.2	
Not very hard	60.0	53.6	71.9	18.5	69.8	67.8	
Self-reported health^d^^,^^e^							
Excellent	21.3	15.1	16.8	4.9	19.3	29.2	<.001
Very good	36.3	32.8	29.4	21.7	36.8	42.2	
Good	29.2	35.7	32.4	46.2	26.1	22.1	
Fair or poor	13.2	16.4	21.4	27.1	17.8	6.5	
Waist circumference, mean (SE), cm^d^^,^^e^	86.4 (0.28)	93.1 (0.54)	77.3 (0.65)	88.2 (0.83)	73.5 (0.52)	85.7 (0.41)	<.001
Smoking status^d^^,^^e^							
Never	58.0	52.9	94.4	66.8	68.6	51.7	<.001
Former	24.7	22.7	3.4	14.4	20.0	32.2	
Current	17.3	24.4	2.2	18.8	11.4	16.2	
Body mass index^d^^,^^f^							
<25	39.8	18.3	76.4	22.8	79.0	42.8	<.001
25-29.9	26.2	28.9	18.4	39.6	15.7	25.3	
≥30	34.0	52.8	5.2	37.6	5.3	31.9	
Alcohol consumption (servings/week, No.)^d^^,^^e^							
None or low (<2)	49.9	57.0	79.1	50.7	56.1	39.6	<.001
Moderate (2-7)	28.6	26.6	14.9	41.6	22.8	30.6	
High (>7)	21.6	16.4	6.0	7.7	21.1	29.8	
Physical Activity Score, mean (SE)^d^^,^^e^^,^^g^	7.6 (0.03)	7.3 (0.06)	7.3 (0.11)	6.8 (0.09)	7.9 (0.10)	8.0 (0.05)	<.001
Medication use through visit 15							
Insulin^d^	4.7	4.4	1.0	2.8	0.5	8.0	<.001
Hypertension^d^	49.4	43.1	35.2	44.8	39.5	67.9	<.001

Abbreviation: NA, not applicable.

^a^ Data are presented as percentage of participants unless otherwise indicated. Most data were multiply imputed (the actual number within each group varies by imputation set [*m* = 10]); thus, exact numerators for the percentages cannot be provided.

^b^ Difference between racial or ethnic groups.

^c^ Variable was fully observed and was not imputed.

^d^ Standard errors between imputation sets were <.05.

^e^ Included as a covariate in subsequent models.

^f^ Calculated as weight in kilograms divided by height in meters squared.

^g^ The Baecke combined physical activity score ranges from 3 to 15; higher scores indicate higher intensity or frequency of work, leisure, or sport activities.

From the screening study, 6521 women (41.5%) were cohort eligible, and of those eligible, 3302 (50.7%) participated; thus, 9174 women (58.5%) were ineligible and left-truncated (excluded) from the cohort. Black women had the highest proportion of left-truncation (61.2%), followed by White (60.0%), Hispanic (59.5%), Chinese (48.1%), and Japanese (44.6%) women.

Nearly a quarter of the women (23.9%) had stage 2 hypertension at baseline and 77.1% during follow-up; thus, 23.9% of hypertension cases were left-censored (ie, prevalent cases). Nearly half of the sample (43.7%) had stage 2 ISH at baseline and 72.5% during follow-up. For metabolic outcomes, 13.5% had IR and 4.6% had diabetes at baseline; 35.9% had IR and 24.2% had diabetes during follow-up. Black and Hispanic women had the largest proportions of all outcomes at baseline (left-censored) and follow-up (Table 2).

**Table 2.** Prevalence of 4 Cardiometabolic Outcomes by Racial or Ethnic Group^a^

Racial or ethnic group	Hypertension, %				Isolated systolic hypertension, %				Insulin resistance, %		Diabetes, %	
	Stage 1^b^		Stage 2^c^		Stage 1^d^		Stage 2^e^					
	Baseline	Follow-up	Baseline	Follow-up	Baseline	Follow-up	Baseline	Follow-up	Baseline	Follow-up	Baseline	Follow-up
All	44.5	89.6	23.9	77.1	44.6	79.4	43.7	72.5	13.5	35.9	4.6	24.2
Black	56.5	97.2	40.1	92.5	64.3	93.5	62.6	89.7	18.7	51.1	8.6	34.8
Chinese	28.4	73.9	13.2	54.6	32.8	61.6	32.8	51.4	11.7	18.4	1.6	15.9
Hispanic	78.3	100	28.0	93.4	26.6	93.9	26.6	88.7	28.7	64.2	5.9	45.1
Japanese	33.8	84.7	11.7	66.2	36.3	67.2	36.3	57.7	4.3	18.2	0.4	12.1
White	35.7	86.6	17.3	70.4	39.5	73.3	38.5	65.3	9.5	27.5	3.2	17.6

^a^ Multiply imputed data from the Study of Women’s Health Across the Nation from 1995 to 2016 (N = 3302). Standard errors between imputation sets were <.05 for all. Median (SE) age at baseline was 46.2 (0.07) years, and median (SE) age at follow-up was 66.5 (0.07) years.

^b^ Classic hypertension (stage 1): systolic blood pressure 130 mm Hg or higher, diastolic blood pressure 80 mm Hg or higher, and/or use of hypertension medication.

^c^ Classic hypertension (stage 2): systolic blood pressure 140 mm Hg or higher, diastolic blood pressure 90 mm Hg or higher, and/or use of hypertension medication.

^d^ Isolated systolic hypertension (stage 1): systolic blood pressure 130 mm Hg or higher and diastolic blood pressure less than 80 mm Hg or use of hypertension medication.

^e^ Isolated systolic hypertension (stage 2): systolic blood pressure 140 mm Hg or higher and diastolic blood pressure less than 90 mm Hg or use of hypertension medication.

A mean of 35.0% of women (range, 32.2%-38.6%) across all outcomes were right-censored. Hispanic women had the highest mean levels of right-censoring (58.5% [range, 52.5%-66.5%]), followed by White (34.3% [range, 32.1%-35.7%]), Japanese (33.3% [range, 26.3%-43.8%]), Chinese (31.7% [range, 22.0%-46.0%]), and Black (30.4% [range, 23.8%-36.4%]) women (eTable 1 in the Supplement).

The estimated difference in median age at onset for stage 2 hypertension was –6.8 years (95% CI, −7.4 to −6.2 years) in models accounting for selection (n = 3302) compared with unadjusted models not accounting for selection (n = 1360). Hispanic women had the largest decrease in estimated median age at onset (–9.7 years; 95% CI, –11.8 to −7.6 years). Incorporating left-censoring was associated with the largest decrease in the estimated median age at onset compared with other mechanisms of selection for all racial or ethnic groups (−6.6 years overall; 95% CI, –6.8 to −6.3 years), with the largest decreases among Hispanic and Black women (eTable 2 in the Supplement). Overall, compared with White women, Black and Hispanic women had a significantly earlier onset of hypertension (Black women: −9.7% [95% CI, −12.5% to −7.0%]; Hispanic women: −9.8% [95% CI, −13.8% to −5.9%]), corresponding to an estimated median age at onset of 5.4 years (95% CI, 5.2-5.6 years) earlier among Black women and 5.5 years (95% CI, 5.2-5.8 years) earlier among Hispanic women compared with White women (Figure and Table 3). Combined, Black and Hispanic women had hypertension a median of 5.0 years (95% CI, 5.4-5.5 years) earlier than White women.

**Figure.**
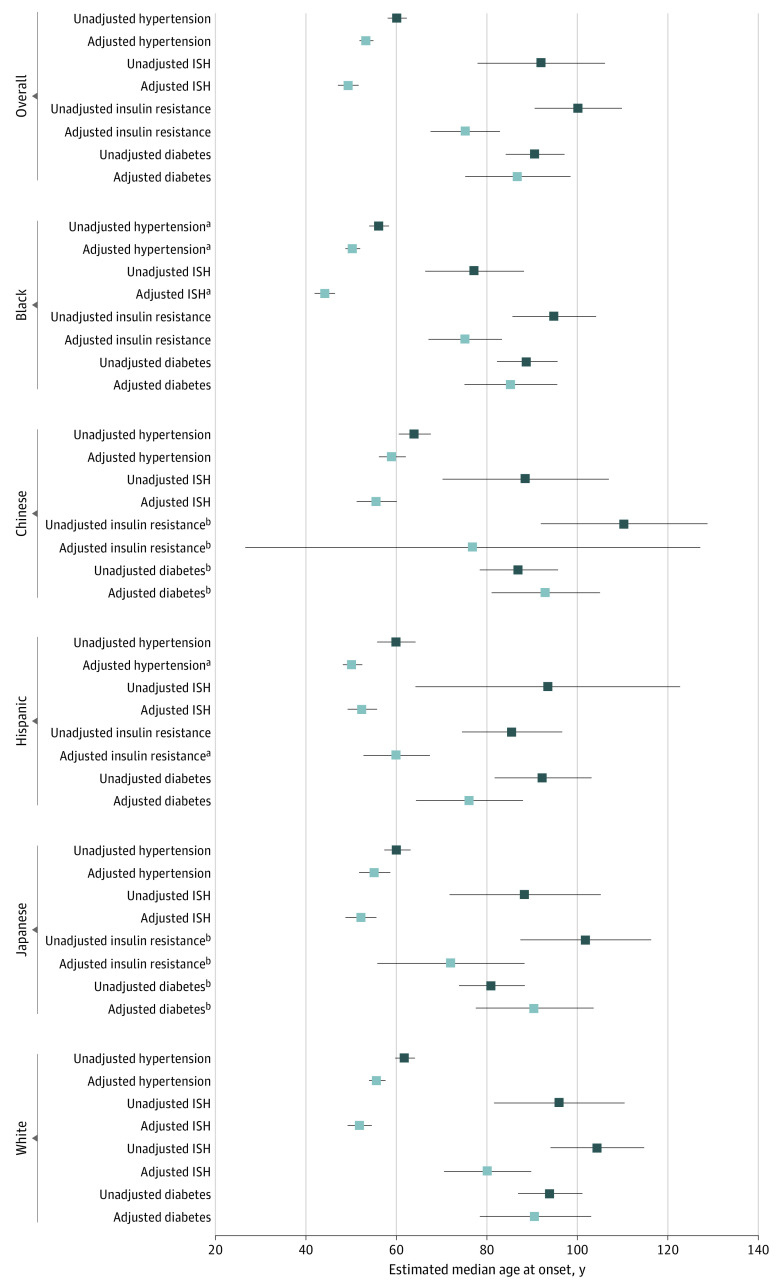
Estimated Median Age at Onset of 4 Cardiometabolic Outcomes Overall and by Racial or Ethnic Group, Unadjusted and Adjusted for Selection Mechanisms Covariates were set as follows: educational level, some college; self-reported health, very good; mean (SE) waist circumference, 86.4 (0.28) cm; smoking status, never; alcohol use, moderate (2-7 drinks per week); and mean (SE) physical activity score, 7.6 (0.03) (score range, 3-15, with higher scores indicating greater intensity or frequency of activity). Data in the unadjusted models are left-censored, right-censored, and unadjusted for left-truncation. The White group is the reference group except for overall estimates. Markers indicate median values and horizontal lines, 95% CIs. ISH indicates isolated systolic hypertension. ^a^Significantly different from White women at *P* < .05. ^b^There were small numbers of cases for Chinese women (approximately 46 with insulin resistance and 40 with diabetes) and Japanese women (approximately 51 with insulin resistance and 34 with diabetes).

**Table 3.** Percentage Difference in Age at Onset and Estimated Median Age at Onset of Stage 2 Hypertension by Racial or Ethnic Group^**a**^

Race or ethnicity	Hypertension				Isolated systolic hypertension			
	Difference in age at onset, % (95% CI)^b^	*P* value	Estimated age at onset^c^		Difference in age at onset, % (95% CI)^b^	*P* value	Estimated age at onset^c^	
			Median, y (95% CI)	Bias (95% CI)^d^			Median, y (95% CI)	Bias (95% CI)^d^
**Unadjusted for selection^e^**								
All	NA	NA	60.2 (58.0 to 62.3)	NA	NA	NA	92.0 (77.9 to 106.1)	NA
Black	−9.3 (−12.0 to −6.5)	.001	56.1 (54.0 to 58.3)	NA	−19.5 (−27.6 to −10.5)	.006	77.3 (66.3 to 88.2)	NA
Chinese	3.5 (−1.4 to 8.5)	.10	64.0 (60.5 to 67.6)	NA	−7.8 (−21.7 to 8.7)	.18	88.6 (70.2 to 106.9)	NA
Hispanic	−3.1 (−9.5 to 3.7)	.19	60.0 (55.7 to 64.2)	NA	−2.7 (−27.1 to 29.9)	.43	93.4 (64.2 to 122.7)	NA
Japanese	−2.7 (−6.6 to 1.3)	.11	60.2 (57.3 to 63.1)	NA	−7.9 (−20.8 to 7.1)	.16	88.4 (71.7 to 105.1)	NA
White	1 [Reference]	NA	61.9 (59.7 to 64.1)	NA	1 [Reference]	NA	96.0 (81.6 to 110.4)	NA
**Adjusted for selection^f^**								
All	NA	NA	53.3 (51.8 to 54.9)	−6.8 (–7.4 to –6.2)	NA	NA	49.3 (47.0 to 51.6)	−42.7 (−54.5 to −30.9)
Black	−9.7 (−12.5 to −7.0)	<.001	50.3 (48.7 to 52.0)	−5.8 (–6.4 to −5.3)	−14.9 (−18.9 to −10.9)	<.001	44.1 (41.9 to 46.4)	−33.1 (–41.8 to −24.5)
Chinese	6.0 (1.8 to 10.2)	.003	59.1 (56.1 to 62.1)	−5.0 (–5.5 to −4.4)	7.3 (0.3 to 14.2)	.02	55.6 (51.2 to 60.0)	−32.9 (–46.9 to −19.0)
Hispanic	−9.8 (−13.8 to −5.9)	<.001	50.3 (48.1 to 52.4)	−9.7 (–11.8 to −7.6)	1.1 (−4.1 to 6.4)	.34	52.4 (49.2 to 55.7)	−41.0 (−67.0 to −15.0)
Japanese	−1.0 (−6.4 to 4.4)	.35	55.2 (51.7 to 58.6)	−5.0 (−5.6 to −4.5)	0.5 (−5.1 to 6.2)	.43	52.1 (48.7 to 55.6)	−36.3 (−49.6 to −23.0)
White	1 [Reference]	NA	55.7 (53.9 to 57.6)	−6.1 (−6.5 to −5.8)	1 [Reference]	NA	51.8 (49.2 to 54.5)	−44.2 (−55.9 to −32.4)

Abbreviation: NA, not applicable.

^a^ Systolic blood pressure 140 mm Hg or higher, diastolic blood pressure 90 mm Hg or higher, and/or use of hypertension medication.

^b^ Calculated as (exp[β] – 1) × 100. Models were adjusted for covariates including educational level, self-reported health, waist circumference, smoking status, alcohol use, and physical activity score.

^c^ Covariates were set to the following: educational level, some college; self-reported health, very good; mean (SE) waist circumference, 86.4 (0.28) cm; smoking status, never; alcohol use, moderate (2-7 drinks per week); and mean (SE) physical activity score, 7.6 (0.03) (score range, 3-15; higher scores indicate higher intensity or frequency of work, leisure, or sport activities).

^d^ Bias is median age at onset in the model adjusted for selection minus median age at onset unadjusted for selection.

^e^ Accelerated failure time survival model was left-censored, right-censored, and unadjusted for left-truncation (hypertension, n = 1360; isolated systolic hypertension, n = 647).

^f^ Weighted interval-censored accelerated failure time survival model with multiply imputed data (hypertension, n = 3302; isolated systolic hypertension, n = 3302).

The estimated difference in median age at onset for stage 2 ISH was –42.7 years (95% CI, –54.5 to –30.9 years) in models accounting for selection (n = 3302) vs unadjusted models not accounting for selection (n = 647). Black and Hispanic women had the largest decrease in estimated median age at onset. Incorporating left-censoring was associated with the largest decrease in estimated median age at onset for all racial or ethnic groups (–46.8 years; 95% CI, −57.6 to –36.0 years), with the largest decreases among Hispanic and White women (eTable 2 in the Supplement). Black women had an earlier estimated onset of ISH compared with White women (–14.9%; 95% CI, −18.9% to −10.9%), corresponding to an estimated median age at onset 7.7 years (95% CI, 7.3-8.1 years) earlier (Figure and Table 3). The estimated median age of onset of ISH was 49.3 years (95% CI, 47.0-51.6 years) overall and 44.1 years (95% CI, 41.9-46.4 years) among Black women.

The difference in estimated median age at onset for IR was –25.0 years (95% CI, −27.0 to −23.0 years) in models accounting for selection (n = 3302) vs unadjusted models not accounting for selection (n = 1620). Accounting for right-censoring was associated with the largest decrease in the estimated median age at onset overall, with the largest decreases among White and Black women (eTable 3 in the Supplement). Overall, compared with White women, the percentage difference in the age at which participants developed IR was –6.2% (95% CI, −12.2% to −0.2%) among Black women and –25.1% (95% CI, −34.2% to −16.0%) among Hispanic women, corresponding to an estimated median age at onset 5.0 years (95% CI, 3.5-6.5 years) earlier for Black women and 20.1 years (95% CI, 17.8-22.4 years) earlier for Hispanic women (Figure and Table 4).

**Table 4.** Percentage Difference in Age at Onset and Estimated Median Age at Onset of Metabolic Outcomes by Racial or Ethnic Group

Race or ethnicity	Insulin resistance				Diabetes			
	Difference in age at onset, % (95% CI)^a^	*P* value	Estimated age at onset^b^		Difference in age at onset, % (95% CI)^a^	*P* value	Estimated age at onset^b^	
			Median, y (95% CI)	Bias (95% CI)^c^			Median, y (95% CI)	Bias (95% CI)^c^
**Unadjusted for selection^d^**								
All	NA	NA	100.2 (90.5 to 109.8)	NA	NA	NA	90.6 (84.1 to 97.1)	NA
Black	−9.1 (−14.6 to −3.3)	.02	94.9 (85.6 to 104.1)	NA	−5.4 (−10.0 to −0.6)	.04	88.9 (82.2 to 95.6)	NA
Chinese^e^	5.7 (−8.6 to 22.1)	.23	110.3 (91.9 to 128.8)	NA	−7.4 (−14.9 to 0.8)	.06	87.1 (78.4 to 95.7)	NA
Hispanic	−18.0 (−27.0 to −8.0)	.011	85.6 (74.5 to 96.6)	NA	−1.7 (−11.4 to 9.2)	.37	92.4 (81.7 to 103.1)	NA
Japanese^e^	−2.4 (−13.3 to 9.8)	.34	101.8 (87.4 to 116.3)	NA	−13.7 (−20.1 to −6.8)	.007	81.1 (73.8 to 88.4)	NA
White	1 [Reference]	NA	104.4 (94.0 to 114.8)	NA	1 [Reference]	NA	94.0 (86.9 to 101.1)	NA
**Adjusted for selection^f^**								
All	NA	NA	75.2 (67.5 to 82.9)	−25.0 (−27.0 to −23.0)	NA	NA	86.8 (75.2 to 98.5)	−3.8 (−9.0 to 1.3)
Black	−6.2 (−12.2 to −0.2)	.02	75.2 (67.1 to 83.3)	−19.7 (−20.8 to −18.6)	−6.0 (−12.1 to 0.1)	.02	85.3 (75.0 to 95.5)	−3.6 (−7.2 to −0.1)
Chinese^e^	−4.2 (−17.9 to 9.5)	.26	76.8 (26.5 to 127.2)	−33.5 (−65.4 to −1.6)	2.5 (−6.3 to 11.3)	.29	93.0 (81.0 to 105.0)	6.0 (2.6 to 9.3)
Hispanic	−25.1 (−34.2 to −16.0)	<.001	60.0 (52.7 to 67.4)	−25.5 (−29.3 to −21.8)	−16.1 (−25.1 to −7.1)	<.001	76.1 (64.3 to 88.0)	−16.3 (−17.4 to −15.2)
Japanese^e^	−10.1 (−27.4 to 7.1)	.11	72.0 (55.7 to 88.3)	−29.8 (−31.6 to −28.0)	−0.2 (−9.0 to 8.6)	.48	90.6 (77.5 to 103.6)	9.5 (3.7 to 15.2)
White	1 [Reference]	NA	80.1 (70.5 to 89.8)	−24.3 (−25.0 to −23.5)	1 [Reference]	NA	90.7 (78.4 to 103.0)	−3.3 (−8.4 to 1.9)

Abbreviation: NA, not applicable.

^a^ Calculated as (exp[β] – 1) × 100. Models were adjusted for covariates including educational level, self-reported health, waist circumference, smoking status, alcohol use, and physical activity score.

^b^ Estimated median age at onset. Covariates were set to the following: educational level, some college; self-reported health, very good; mean (SE) waist circumference, 86.4 (0.28) cm; smoking status, never; alcohol use, moderate (2-7 drinks per week); and mean (SE) physical activity score, 7.6 (0.03) (score range, 3-15; higher scores indicate higher intensity or frequency of work, leisure, or sport activities).

^c^ Bias is median age at onset in the model adjusted for selection minus median age at onset unadjusted for selection.

^d^ Accelerated failure time survival model was left-censored, right-censored, and unadjusted for left truncation (insulin resistance, n = 1620; diabetes, n = 1893).

^e^ There were small numbers of cases for Chinese women (approximately 46 with insulin resistance and 40 with diabetes) and Japanese women (approximately 51 with insulin resistance and 34 with diabetes).

^f^ Weighted interval-censored accelerated failure time survival model with multiply imputed data (insulin resistance, n = 3302; diabetes, n = 3302).

There was no overall statistically significant decrease in estimated median age at onset for diabetes after considering all selection mechanisms (adjusted [n = 3302] vs unadjusted [n = 1893]). However, there were statistically significant decreases by race or ethnicity, with Black women having a difference in age at diabetes onset of –6.0% (95% CI, −12.1% to 0.1%) and Hispanic women a difference of –16.1% (95% CI, −25.1% to −7.1%) compared with White women, corresponding to estimated median ages at onset 5.4 years (95% CI, 3.4-7.5 years) and 14.6 years (95% CI, 14.1-15.1 years) earlier, respectively (Figure, Table 4, and eTable 3 in the Supplement). Combined, Black and Hispanic women had metabolic outcomes (diabetes and IR) a median of 11.3 years (95% CI, 9.7-12.9 years) earlier than White women.

## Discussion

This study provides an assessment of the potential magnitude of racial or ethnic differences in age at onset of hypertension, ISH, IR, and diabetes in midlife women in the US. Hypertension occurred a median of 5.4 years earlier among Black women and 5.5 years earlier among Hispanic women compared with White women. Isolated systolic hypertension was highly prevalent in Black women, with an estimated median age at onset 7.7 years earlier than among White women. Metabolic outcomes occurred a median of 11.3 years earlier for Black and Hispanic women compared with White women. In this study, addressing all selection mechanisms was associated with a mean 20-year decrease in the estimated median age at onset for all outcomes across racial or ethnic groups.

The finding of earlier onset of cardiometabolic outcomes for Black and Hispanic women compared with White women adds to a growing body of research^4,5,6,27^ showing that racial and ethnic minoritized groups experience earlier average onset of a variety of adverse health outcomes. Cardiometabolic diseases are the main factors associated with a shorter life span,^1^ and racial and ethnic differences exist in the prevalence of cardiometabolic outcomes, including hypertension, diabetes, metabolic syndrome, and cardiovascular disease.^3,4,28,29,30^ Racial and ethnic disparities can also be conceptualized from a life-course perspective^5,7,8,9^ as the differences in the timing or average age at onset of a particular outcome. Results suggest that weathering or accelerated health declines^4,5^ occur among Black and Hispanic women independent of socioeconomic and health behavior factors. Of importance, ISH has been estimated previously to begin around age 60 years for women in the US^13,14^ and is associated with increased rates of cardiovascular and kidney diseases.^14^ In this study, the estimated overall median age at onset of stage 2 ISH was 49.3 years, and for Black women, it was as early as 41.9 to 46.4 years of age, suggesting that ISH may be an important target for intervention in early midlife women, especially for Black women. Earlier occurrence of these major cardiometabolic factors may be associated with earlier disability and mortality, ultimately driving persistent racial and ethnic differences in life span.^1,2,31^

This study corrected for multiple forms of selection bias. The results suggest that selection bias has the potential to underestimate the age of onset of cardiometabolic outcomes overall, and particularly for racial and ethnic minoritized groups. Selection bias can arise from multiple sources in a cohort study.^11,22^ Left-truncation occurs when individuals who have already had the outcome of interest are not included in the study. Left-censoring occurs when individuals who have already had the outcome of interest are included but are then excluded from analyses due to having the outcome. Right-censoring occurs when the outcome is not observed due to loss to follow-up or missing variables.^10^ Bias can occur if the probability of selection through any of these mechanisms differs across relevant characteristics, such as race and ethnicity, that might influence the question of interest.^12,32^ Much effort has focused on mitigating the effect of right-censoring,^12,33^ but less research has focused on mitigating potential bias caused by left-truncation and censoring^10,32,34^—the main sources of selection bias likely affected by weathering.

Adjustment for selection into the cohort (ie, left-truncation and censoring) in this study was the factor most associated with a decrease in the estimated age at onset of cardiometabolic outcomes, especially hypertension outcomes. Based on age-specific prevalence estimates, the mean age at onset of hypertension (45-64 years) is earlier than that of metabolic outcomes (55-74 years).^4,13,14,15^ Consequently, hypertension was highly prevalent at the SWAN baseline (42-52 years of age), leading to a large amount of left-censoring while also signaling that there are likely high levels of left-truncation for hypertension (ie, individuals with hypertension are excluded from the study). Therefore, incorporating left-censored cases and accounting for left-truncation were associated with a decrease in the estimated median age at onset of hypertension. Although the outcomes in this study were not variables used to select the SWAN cohort (ie, menopausal stage), results for hypertension were similar to those of a simulation conducted by Cain et al^10^ that showed that accounting for left-truncation and censoring was associated with decreases in the age at onset of the outcome of interest.

### Limitations

This study has limitations. Chinese and Japanese women had a low prevalence and incidence of metabolic outcomes, leading to imprecision; thus, estimates should be interpreted with caution. It is possible that women taking insulin did not have IR, especially women with type 1 diabetes; however, less than 1% of those with diabetes have type 1,^35^ and many have overweight or obesity and have IR.^36^ In SWAN, the body mass index of women with insulin-treated diabetes did not differ from that of women not treated with insulin with HOMA-IR values greater than 5.9 mLU/L. Therefore, it is unlikely that misclassification of IR among insulin-treated women with diabetes would change the results. There was a substantial amount of right-censoring in this sample, especially among Hispanic women. Multiple imputation assumes that data are missing at random^21^; therefore, some bias may remain in the estimates, likely causing an underestimate of racial or ethnic disparities, especially for Hispanic women. However, results were robust across nonimputed and imputed data (eTables 2 and 3 in the Supplement). Analyses corrected for left-truncation from the screening study to the cohort study; however, although some probability-based recruiting procedures were used, we cannot assume that there was no additional bias stemming from selection into the screening study. Given that studies such as the cross-sectional screening study often are not generalizable to the entire relevant population,^37^ future work should consider additionally correcting for selection into the screening study. This study also evaluated racial or ethnic disparities accounting for some individual-level socioeconomic and behavioral factors, but these factors are not inclusive of all factors that may be associated with racial or ethnic disparities. Further work should evaluate how structural and interpersonal racism contributes to cardiometabolic disparities while accounting for selection biases.

## Conclusions

In this longitudinal cohort study of midlife women, the results suggest that hypertension, and particularly ISH for Black women, are important first targets for intervention as early as age 30 years, whereas intervention for diabetes and IR may need to first target Hispanic women aged 40 to 50 years. Considering racial and ethnic disparities from a life-course perspective allows evaluation of differences in weathering. Understanding age at onset can guide the timing of clinical interventions to the appropriate life stages and time points for primary prevention. Furthermore, the results suggest that research aimed at evaluating disparities should consider the impact of selection biases, especially stemming from the understudied differential inclusion into studies (left-truncation and censoring). In this study, Hispanic and Black women had the highest proportions of left-truncation and censoring indicative of weathering; thus, incorporating and accounting for these sources of bias was associated with lower predicted ages at cardiometabolic onset, especially for these groups. Further work should consider the consequences of selection biases for other important outcomes of interest, especially those that may be associated with racial and ethnic differences in longevity. Fully considering the extent of selection bias can inform researchers and clinicians about the optimal timing for interventions, particularly for high-risk groups that require renewed and continued attention to early structural factors that shape life-course health and aging.

## Notes

Supplement. **eTable 1.** Missing Patterns in the Study of Women’s Health Across the Nation (SWAN) Across 4 Cardiometabolic Outcomes, 1995-2016 (N = 3302) **eTable 2.** Percentage Difference in Age at Onset and Estimated Median Age at Onset of Hypertension (Stage 2) by Racial or Ethnic Group in the Study of Women’s Health Across the Nation **eTable 3.** Percentage Difference in Age at Onset and Estimated Median Age at Onset of Metabolic Outcomes by Racial or Ethnic Group in the Study of Women’s Health Across the Nation
