# Supplementary material for: Study Selection Bias and Racial or Ethnic Disparities in Estimated Age at Onset of Cardiometabolic Disease Among Midlife Women in the US
Source: JAMA Netw Open. 2022 Nov 7;5(11):e2240665. doi: 10.1001/jamanetworkopen.2022.40665 (PMC9641536; doi:10.1001/jamanetworkopen.2022.40665)
Supplement: Supplement. — eTable 1. Missing Patterns in the Study of Women’s Health Across the Nation (SWAN) Across 4 Cardiometabolic Outcomes, 1995-2016 (N = 3302) eTable 2. Percentage Difference in Age at Onset and Estimated Median Age at Onset of Hypertension (Stage 2) by Racial or Ethnic Group in the Study of Women’s Health Across the Nation eTable 3. Percentage Difference in Age at Onset and Estimated Median Age at Onset of Metabolic Outcomes by Racial or Ethnic Group in the Study of Women’s Health Across the Nation [file jamanetwopen-e2240665-s001.pdf]

Supplementary Online Content

Reeves A, Elliott MR, Lewis TT, Karvonen-Gutierrez CA, Herman WH, Harlow SD. Study selection bias and racial or ethnic disparities in estimated age at onset of cardiometabolic disease among midlife women in the US. *JAMA Netw Open*. 2022;5(11):e2240665. doi:10.1001/jamanetworkopen.2022.40665

eTable 1. Missing Patterns in the Study of Women's Health Across the Nation (SWAN) Across 4 Cardiometabolic Outcomes, 1995-2016 (N = 3302)
eTable 2. Percentage Difference in Age at Onset and Estimated Median Age at Onset of Hypertension (Stage 2) by Racial or Ethnic Group in the Study of Women's Health Across the Nation
eTable 3. Percentage Difference in Age at Onset and Estimated Median Age at Onset of Metabolic Outcomes by Racial or Ethnic Group in the Study of Women's Health Across the Nation

This supplementary material has been provided by the authors to give readers additional information about their work.

© 2022 Reeves A et al. *JAMA Network Open*.

eTable 1. Missing Patterns in the Study of Women's Health Across the Nation (SWAN) Across 4 Cardiometabolic Outcomes, 1995-2016 (N = 3302)

	Observed[a]	Missing Interval	Loss to Follow Up
	% (n)	% (n)	% (n)
Hypertension			
Overall	67.9 (2241)	8.5 (279)	23.7 (782)
Racial/Ethnic Group			
Black	76.3 (713)	8.4 (78)	15.3 (143)
Chinese	70.8 (177)	4.8 (12)	24.4 (61)
Hispanic	45.5 (130)	17.5 (50)	37.1 (106)
Japanese	70.1 (197)	4.6 (13)	25.3 (71)
White	66.0 (1024)	8.1 (126)	25.9 (401)
Isolated Systolic HTN			
Overall	64.6 (2132)	8.9 (293)	26.6 (877)
Racial/Ethnic Group			
Black	72.4 (676)	9.5 (89)	18.1 (169)
Chinese	70.4 (176)	4.4 (11)	25.2 (63)
Hispanic	33.6 (96)	21.7 (62)	44.8 (128)
Japanese	66.9 (188)	5.0 (14)	28.1 (79)
White	64.2 (996)	7.5 (117)	28.2 (438)
Insulin Resistance			
Overall	61.5 (2029)	8.5 (280)	30.1 (993)
Racial/Ethnic Group			
Black	63.6 (594)	13.3 (124)	23.1 (216)
Chinese	54.0 (135)	7.6 (19)	38.4 (96)
Hispanic	47.6 (136)	11.2 (32)	41.3 (118)
Japanese	56.2 (158)	6.1 (17)	37.7 (106)
White	64.9 (1006)	5.7 (88)	29.5 (457)
Diabetes			
Overall	66.2 (2186)	1.9 (64)	31.9 (1052)
Racial/Ethnic Group			
Black	66.3 (619)	1.9 (18)	31.8 (297)
Chinese	78.0 (195)	0.0 (0)	22.0 (55)
Hispanic	39.2 (112)	9.4 (27)	51.4 (147)
Japanese	73.7 (207)	0.0 (0)	26.3 (74)
White	67.9 (1053)	1.2 (19)	30.9 (479)

Note: HTN = hypertension.
[a]Observed fully or carried forward (due to no change in status through interval).

© 2022 Reeves A et al. *JAMA Network Open*.

eTable 2. Percentage Difference in Age at Onset and Estimated Median Age at Onset of Hypertension (Stage 2) by Racial or Ethnic Group in the Study of Women's Health Across the Nation

	**Hypertension**										**Isolated Systolic HTN**									
	Diff in Age of Onset[e]				Predicted Median Age of Onset[f]						Diff in Age of Onset[e]				Predicted Median Age of Onset[f]					
	%	LCI	UCI	p-value	Median	LCI	UCI	Bias[g]	LCI	UCI	%	LCI	UCI	p-value	Median	LCI	UCI	Bias[g]	LCI	UCI
**Model 1 - Unadjusted[a]**																				
Overall	*NA*				60.2	58.0	62.3	*NA*			*NA*				92.0	77.9	106.1	*NA*		
Racial/Ethnic Group																				
Black	-9.3	-12.0	-6.5	0.001	56.1	54.0	58.3				-19.5	-27.6	-10.5	0.006	77.3	66.3	88.2			
Chinese	3.5	-1.4	8.5	0.10	64.0	60.5	67.6				-7.8	-21.7	8.7	0.18	88.6	70.2	106.9			
Hispanic	-3.1	-9.5	3.7	0.19	60.0	55.7	64.2	*NA*			-2.7	-27.1	29.9	0.43	93.4	64.2	122.7	*NA*		
Japanese	-2.7	-6.6	1.3	0.11	60.2	57.3	63.1				-7.9	-20.8	7.1	0.16	88.4	71.7	105.1			
White	*reference*				61.9	59.7	64.1				*reference*				96.0	81.6	110.4			
**Model 2 - Incorporating Left Censored[b]**																				
Overall	*NA*				53.6	51.3	55.9	-6.6	-6.8	-6.3	*NA*				45.2	41.9	48.5	-46.8	-36.0	-57.6
Racial/Ethnic Group																				
Black	-14.9	-17.9	-11.9	0.000	48.5	46.2	50.8	-7.6	-7.7	-7.6	-18.4	-23.1	-13.5	0.001	39.6	36.4	42.7	-37.7	-29.9	-45.5
Chinese	5.2	-1.0	11.8	0.07	60.0	55.8	64.1	-4.1	-4.7	-3.5	8.9	-1.7	20.5	0.07	52.8	46.7	59.0	-35.7	-23.5	-48.0
Hispanic	-16.7	-22.4	-10.5	0.003	47.5	43.9	51.1	-12.5	-11.8	-13.1	-12.6	-23.2	-0.5	0.05	42.4	36.6	48.2	-51.0	-27.6	-74.5
Japanese	-1.5	-6.6	3.8	0.28	56.1	52.6	59.6	-4.1	-4.6	-3.5	-4.4	-12.8	4.7	0.18	46.4	41.4	51.3	-42.0	-30.3	-53.8
White	*reference*				57.0	54.5	59.5	-4.9	-5.2	-4.6	*reference*				48.5	44.9	52.1	-47.5	-36.6	-58.3
**Model 3 - Additionally Accounting for Right Censoring[c]**																				
Overall	*NA*				54.4	53.1	55.6	-5.8	-4.9	-6.7	*NA*				50.3	48.3	52.2	-41.7	-29.7	-53.8
Racial/Ethnic Group																				
Black	-12.1	-14.1	-10.2	0.000	50.0	48.7	51.3	-6.1	-5.2	-7.0	-16.0	-19.4	-12.6	0.000	44.5	42.5	46.5	-32.8	-23.8	-41.7
Chinese	4.9	1.5	8.2	0.003	59.7	57.4	62.0	-4.3	-3.1	-5.6	5.9	0.1	11.6	0.03	56.1	52.4	59.8	-32.5	-17.8	-47.2
Hispanic	-10.0	-13.2	-6.8	0.000	51.2	49.4	53.1	-8.7	-6.3	-11.2	-0.6	-5.9	4.6	0.41	52.6	49.5	55.8	-40.8	-14.7	-66.9
Japanese	-0.9	-3.9	2.1	0.28	56.4	54.5	58.4	-3.8	-2.8	-4.7	-2.2	-7.4	3.0	0.20	51.8	48.7	54.9	-36.6	-23.0	-50.2
White	*reference*				56.9	55.6	58.3	-5.0	-4.1	-5.8	*reference*				53.0	50.8	55.2	-43.0	-30.8	-55.3
**Model 4 - Additionally Adjusted for Left Truncation[d]**																				
Overall	*NA*				53.3	51.8	54.9	-6.8	-6.2	-7.4	*NA*				49.3	47.0	51.6	-42.7	-30.9	-54.5
Racial/Ethnic Group																				
Black	-9.7	-12.5	-7.0	0.000	50.3	48.7	52.0	-5.8	-5.3	-6.4	-14.9	-18.9	-10.9	0.000	44.1	41.9	46.4	-33.1	-24.5	-41.8
Chinese	6.0	1.8	10.2	0.003	59.1	56.1	62.1	-5.0	-4.4	-5.5	7.3	0.3	14.2	0.02	55.6	51.2	60.0	-32.9	-19.0	-46.9
Hispanic	-9.8	-13.8	-5.9	0.000	50.3	48.1	52.4	-9.7	-7.6	-11.8	1.1	-4.1	6.4	0.34	52.4	49.2	55.7	-41.0	-15.0	-67.0
Japanese	-1.0	-6.4	4.4	0.35	55.2	51.7	58.6	-5.0	-5.6	-4.5	0.5	-5.1	6.2	0.43	52.1	48.7	55.6	-36.3	-23.0	-49.6
White	*reference*				55.7	53.9	57.6	-6.1	-5.8	-6.5	*reference*				51.8	49.2	54.5	-44.2	-32.4	-55.9

Note: HTN = Hypertension, Diff = Difference.

[a]Accelerated failure time survival model that is left-censored, right-censored and unadjusted for left-truncation (n_{HTN} = 1360, n_{ISH} = 647).

[b]Interval censored accelerated failure time survival model to incorporate left censored cases (n_{HTN} = 2084, n_{ISH} = 1986).

[c]Interval censored accelerated failure time survival model with multiply imputed data (n_{HTN} = 3302, n_{ISH} = 3302).

[d]Weighted interval censored accelerated failure time survival model with multiply imputed data (n_{HTN} = 3302, n_{ISH} = 3302).

[e]Percent difference in age of onset calculated as (exp(β)-1)*100. Models adjusted for covariates including educational level, self-reported health, waist circumference, smoking status, alcohol use and physical activity score.

[f]Covariates set to: educational level (some college), self-reported health (very good), waist circumference (86.34 cm), smoking status (never), alcohol use (moderate) and physical activity score (7.65).

[g]Bias is median age of onset in model adjusted for selection minus median age of onset unadjusted for selection (model 1).

© 2022 Reeves A et al. *JAMA Network Open*.

eTable 3. Percentage Difference in Age at Onset and Estimated Median Age at Onset of Metabolic Outcomes by Racial or Ethnic Group in the Study of Women's Health Across the Nation

	**Insulin Resistance**										**Diabetes**									
	Diff in Age of Onset[e]				Predicted Median Age of Onset[f]						Diff in Age of Onset[e]				Predicted Median Age of Onset[f]					
	%	LCI	UCI	p-value	Median	LCI	UCI	Bias[g]	LCI	UCI	%	LCI	UCI	p-value	Median	LCI	UCI	Bias[g]	LCI	UCI
**Model 1 - Unadjusted[a]**																				
Overall	*NA*				100.2	90.5	109.8	*NA*			*NA*				90.6	84.1	97.1	*NA*		
Racial/Ethnic Group																				
Black	-9.1	-14.6	-3.3	0.02	94.9	85.6	104.1	*NA*			-5.4	-10.0	-0.6	0.04	88.9	82.2	95.6	*NA*		
Chinese[h]	5.7	-8.6	22.1	0.23	110.3	91.9	128.8				-7.4	-14.9	0.8	0.06	87.1	78.4	95.7			
Hispanic	-18.0	-27.0	-8.0	0.011	85.6	74.5	96.6				-1.7	-11.4	9.2	0.37	92.4	81.7	103.1			
Japanese[h]	-2.4	-13.3	9.8	0.34	101.8	87.4	116.3				-13.7	-20.1	-6.8	0.007	81.1	73.8	88.4			
White	*reference*				104.4	94.0	114.8				*reference*				94.0	86.9	101.1			
**Model 2 - Incorporating Left Censored[b]**																				
Overall	*NA*				101.1	90.5	111.7	0.9	-0.1	1.8	*NA*				101.5	92.0	111.0	10.9	7.9	13.9
Racial/Ethnic Group																				
Black	-10.1	-16.4	-3.3	0.02	96.1	85.7	106.5	1.3	0.1	2.4	-8.4	-14.1	-2.3	0.02	98.1	88.6	107.7	9.2	6.3	12.1
Chinese[h]	9.1	-7.7	28.9	0.16	116.6	94.7	138.6	6.3	2.7	9.8	-3.2	-13.8	8.6	0.29	103.6	89.5	117.8	16.6	11.1	22.1
Hispanic	-27.2	-35.7	-17.6	0.003	77.8	66.9	88.7	-7.7	-7.5	-8.0	-13.2	-23.2	-1.8	0.04	93.0	80.4	105.7	0.6	-1.3	2.6
Japanese[h]	4.9	-10.2	22.6	0.27	112.2	92.1	132.2	10.3	4.8	15.9	-12.2	-21.2	-2.3	0.03	94.0	82.2	105.8	12.9	8.4	17.5
White	*reference*				106.9	95.3	118.5	2.5	1.2	3.8	*reference*				107.1	96.5	117.8	13.1	9.6	16.7
**Model 3 - Additionally Accounting for Right Censoring[c]**																				
Overall	*NA*				78.0	73.3	82.7	-22.2	-17.2	-27.1	*NA*				88.9	84.4	93.5	-1.7	0.2	-3.6
Racial/Ethnic Group																				
Black	-11.2	-15.2	-7.2	0.000	74.3	69.9	78.7	-20.6	-15.7	-25.4	-7.3	-10.9	-3.7	0.000	86.3	81.5	91.1	-2.6	-0.8	-4.5
Chinese[h]	-5.2	-17.2	6.7	0.18	79.4	37.9	120.9	-31.0	-54.0	-7.9	-3.5	-10.3	3.3	0.15	89.8	82.6	97.0	2.7	4.2	1.3
Hispanic	-25.8	-32.2	-19.4	0.000	62.1	56.2	68.0	-23.5	-18.3	-28.6	-13.7	-19.2	-8.1	0.000	80.3	74.7	85.9	-12.1	-7.0	-17.2
Japanese[h]	-4.2	-12.7	4.3	0.16	80.2	68.4	92.0	-21.7	-19.0	-24.4	-5.0	-11.7	1.7	0.07	88.4	81.3	95.4	7.3	7.5	7.1
White	*reference*				83.7	77.8	89.6	-20.7	-16.3	-25.2	*reference*				93.0	87.8	98.3	-0.9	0.9	-2.8
**Model 4 - Additionally Adjusted for Left Truncation[d]**																				
Overall	*NA*				75.2	67.5	82.9	-25.0	-23.0	-27.0	*NA*				86.8	75.2	98.5	-3.8	-9.0	1.3
Racial/Ethnic Group																				
Black	-6.2	-12.2	-0.2	0.02	75.2	67.1	83.3	-19.7	-18.6	-20.8	-6.0	-12.1	0.1	0.02	85.3	75.0	95.5	-3.6	-7.2	-0.1
Chinese[h]	-4.2	-17.9	9.5	0.26	76.8	26.5	127.2	-33.5	-65.4	-1.6	2.5	-6.3	11.3	0.29	93.0	81.0	105.0	6.0	2.6	9.3
Hispanic	-25.1	-34.2	-16.0	0.000	60.0	52.7	67.4	-25.5	-21.8	-29.3	-16.1	-25.1	-7.1	0.000	76.1	64.3	88.0	-16.3	-17.4	-15.2
Japanese[h]	-10.1	-27.4	7.1	0.11	72.0	55.7	88.3	-29.8	-31.6	-28.0	-0.2	-9.0	8.6	0.48	90.6	77.5	103.6	9.5	3.7	15.2
White	*reference*				80.1	70.5	89.8	-24.3	-23.5	-25.0	*reference*				90.7	78.4	103.0	-3.3	-8.4	1.9

Note: Diff = Difference.

[a]Accelerated failure time survival model that is left censored, right censored and unadjusted for left truncation (n_{IR} = 1620, $n_{Diabetes}$ = 1893).

[b]Interval censored accelerated failure time survival model to incorporate left censored cases (n_{IR} = 1887, $n_{Diabetes}$ = 2033).

[c]Interval censored accelerated failure time survival model with multiply imputed data (n_{IR} = 3302, $n_{Diabetes}$ = 3302).

[d]Weighted interval censored accelerated failure time survival model with multiply imputed data (n_{IR} = 3302, $n_{Diabetes}$ = 3302).

[e]Percent difference in age of onset calculated as (exp(β)-1)*100. Models adjusted for covariates including educational level, self-reported health, waist circumference, smoking status, alcohol use and physical activity score.

[f]Covariates set to: educational level (some college), self-reported health (very good), waist circumference (86.34 cm), smoking status (never), alcohol use (moderate) and physical activity score (7.65).

[g]Bias is median age of onset in model adjusted for selection minus median age of onset unadjusted for selection (model 1).

[h]Small number of cases for Chinese (n with insulin resistance ~46, n with diabetes ~40) and Japanese (n with insulin resistance ~51, n with diabetes ~34) women.

© 2022 Reeves A et al. *JAMA Network Open*.
